# Development and Analytical Evaluation of Urine-Based Test for Detection of Pancreatic Cancer

**DOI:** 10.3390/diagnostics16142215

**Published:** 2026-07-15

**Authors:** Maria Dekarz, Małgorzata Gawrońska, Bryan Mierzwa, Jakub Muchowski, Patrycja Ogonowska, Marzena Skowrońska, Katarzyna Stempnakowska

**Affiliations:** URTESTE S.A., Starodworska 1, 80-137 Gdańsk, Poland; maria.dekarz@urteste.eu (M.D.); bryan.mierzwa@urteste.eu (B.M.); jakub.muchowski@urteste.eu (J.M.);

**Keywords:** pancreatic ductal adenocarcinoma, PDAC, urine-based assay, proteolytic activity, chromogenic substrate, qualitative diagnostic test, analytical validation, interference testing

## Abstract

**Background/Objectives**: Pancreatic ductal adenocarcinoma (PDAC) remains a highly lethal malignancy lacking non-invasive functional diagnostic tools. The objective of this study was to develop and analytically validate a qualitative chromogenic urine assay (Panuri) detecting proteolytic activity associated with pancreatic cancer. **Methods**: This study reports on the analytical validation of a prototype configuration of the Panuri assay. The assay is based on enzymatic hydrolysis of synthetic peptide substrates measured spectrophotometrically at 410 nm. Results were interpreted qualitatively using predefined OD/h cut-off values (R1: 0.002206; R2: 0.003343; R3: 0.000766). Analytical validation included determination of sensitivity and specificity, precision assessment, and interference testing with microorganisms (10^7^ CFU/mL) and selected endogenous substances. **Results**: At the established OD/h cut-off, the assay demonstrated 89% sensitivity (95% CI: 78.5–94.9%) and 75% specificity (95% CI: 64.8–83.5%). Precision studies demonstrated repeatability, within-laboratory precision, and inter-laboratory reproducibility, with coefficients of variation below 10%. Interference from endogenous substances was limited under testing conditions, whereas high microbial contamination induced classification shifts. Further analytical studies are ongoing to assess the impact of exogenous interferents, including drugs and supplements, in accordance with extended interference evaluation requirements. **Conclusions**: The Panuri assay demonstrates predefined analytical performance characteristics for qualitative detection of proteolytic activity in the urine under the conditions evaluated in this study.

## 1. Introduction

Pancreatic cancer, which has one of the highest mortality rates among all malignancies, is the sixth leading cause of cancer-related death worldwide [1]. Due to its poor prognosis, the number of deaths (467,409) is nearly equal to the number of new cases (510,992) [2]. Among malignant pancreatic neoplasms, 90% are pancreatic ductal adenocarcinoma (PDAC) [3,4]. Most patients have no obvious symptoms during disease development. Therefore, early detection is difficult and by the time of diagnosis pancreatic cancer often presents at an advanced stage and has often spread to other parts of the body. It is still a highly lethal gastrointestinal cancer with a low 5-year survival rate [5]. Only 15–20% of patients with pancreatic cancer present with resectable disease at the time of diagnosis [6,7]. Moreover, epidemiological data suggest that after clinical manifestation, pancreatic cancer may progress from stage T1 to T4 within approximately one year [8]. Taken together, these findings indicate that pancreatic cancer diagnosis remains a major clinical challenge, while the rapid progression of the disease highlights the pressing need for the development of novel, non-invasive and easily accessible diagnostic approaches.

Methods for diagnosis of pancreatic cancer can be broadly divided into two main categories: biomarker assessment and imaging techniques. However, both approaches remain insufficient for reliable detection at a preclinical stage. CA19-9 (carbohydrate antigen 19-9), the most widely used and FDA-approved serum biomarker, shows variable sensitivity (41–86%) and specificity (33–100%) and performs particularly poorly with early-stage and small-diameter tumors [9,10]. Consequently, it is not recommended as a screening tool by the American Society of Clinical Oncology (ASCO) and the European Group of Tumoral Markers (EGTM), and its role is largely adjunctive to imaging [11,12]. Similarly, CEA (carcinoembryonic antigen) demonstrates limited sensitivity and insufficient specificity in pancreatic cancer and is not recommended for routine diagnostic use [10]. Imaging modalities, including CT (Computed Tomography), MRI (Magnetic Resonance Imaging) and EUS (Endoscopic Ultrasound), constitute the cornerstone of structural assessment, yet their performance declines for small or early-stage lesions. CT sensitivity decreases to approximately 63–77% for tumors < 2 cm [13], and MRI does not provide a substantial overall advantage over CT in pancreatic cancer detection [14]. While EUS improved detection rate compared with CT and MRI in early stages [15], it remains invasive and operator dependent [16]. Even advanced techniques such as EUS-FNA (Endoscopic Ultrasound Fine-Needle Aspiration) and EUS-FNB (Endoscopic Ultrasound Fine-Needle Biopsy), despite high diagnostic accuracy in established lesions, are primarily confirmatory rather than screening tools [17,18].

Collectively, these limitations demonstrate that current biomarker- and imaging-based strategies are insufficient for reliable non-invasive detection and diagnostic stratification of pancreatic cancer, highlighting the need for novel, non-invasive, accessible and cost-effective diagnostic tools.

The growing interest in urinary biomarkers is based on the unique physiological characteristics of urine as a biological sample. Unlike blood and other components of the internal environment, urine is not subject to strict homeostatic regulation. Homeostatic mechanisms continuously act to maintain the stability of blood composition by buffering and removing physiological and pathological changes, thereby masking many early disease-related alterations. In contrast, urine serves as a reservoir for metabolic and molecular changes occurring throughout the body, allowing the accumulation of biological information that may not be detectable in blood. Consequently, subtle pathological processes can be reflected in urine at an earlier stage of disease development, often before the appearance of clinical symptoms, imaging abnormalities, or detectable changes in circulating biomarkers. These features make urine a particularly informative source for biomarker discovery [19,20,21].

Proteomic analyses have demonstrated that urine contains thousands of proteins and peptides, including molecules derived from pathological processes such as cancer. In patients with various malignancies, distinct alterations in the urinary proteomic profile have been reported, including changes in protein abundance and the presence of characteristic peptide fragments. These proteomic patterns reflect systemic biological processes associated with tumor development and progression [22]. A central mechanism underlying these alterations is the dysregulation of proteolytic enzymes in cancer. Proteases play a fundamental role in tumor biology by mediating extracellular matrix degradation, remodeling of the tumor microenvironment, tumor cell invasion, and angiogenesis. Increased expression and activity of tumor-associated proteases, including matrix metalloproteinases (MMPs), serine proteases, and cysteine proteases, have been documented across multiple cancer types and are frequently associated with tumor aggressiveness and disease progression. Enhanced proteolysis leads to the generation of specific protein fragments that contribute to pathological tissue remodeling and shape the cancer-associated proteomic landscape [23]. Proteases and their cleavage products may enter the circulation and subsequently undergo renal filtration, resulting in their detectable presence and enzymatic activity in urine. Consequently, urinary proteolytic activity can mirror protease-driven processes occurring within the tumor and its microenvironment. This provides a biologically plausible link between cancer-associated proteolysis and measurable urinary enzymatic activity, supporting the rationale for exploring urinary proteolytic signatures as non-invasive biomarkers in oncology [24].

Among solid malignancies, pancreatic ductal adenocarcinoma (PDAC) has emerged as an important target for urine-based biomarker research due to the urgent need for effective non-invasive tools enabling earlier diagnosis. Several studies have demonstrated that urine contains diagnostically relevant molecular signatures associated with pancreatic carcinogenesis. One of the most extensively investigated approaches is the urinary protein biomarker panel comprising LYVE-1 (lymphatic vessel endothelial HA receptor), TFF1 (trefoil factor family), and REG1A (regenerating islet-derived 1 alpha). In a multicenter study conducted by Radon et al., urinary concentrations of all three proteins were significantly elevated in PDAC patients compared with both healthy controls and patients with chronic pancreatitis. Importantly, increased biomarker levels were also observed in patients with stage I–II disease and even in stage I–IIA tumors without lymph node involvement, indicating their potential diagnostic relevance in PDAC, including less advanced disease stages. Furthermore, these biomarkers were able to distinguish PDAC from chronic pancreatitis, a clinically important differential diagnosis [25].

In addition to protein biomarkers, urinary non-coding RNAs have attracted considerable interest as potential diagnostic tools. Debernardi et al. demonstrated that several urinary microRNAs, including miR-30e, miR-143, miR-204, and miR-223, were significantly overexpressed in patients with early-stage PDAC [26]. Notably, some of these miRNAs had previously been identified in pancreatic tumor tissues and pancreatic cyst fluid, supporting their biological association with pancreatic neoplasia [27,28]. These findings suggest that urinary miRNA profiles may reflect early molecular alterations associated with tumor initiation and progression, although further validation in larger patient cohorts is required.

More recently, metabolomic approaches have expanded the spectrum of urinary biomarkers through the analysis of volatile organic compounds (VOCs). VOC profiling using field asymmetric ion mobility spectrometry (FAIMS) demonstrated the ability to differentiate PDAC patients from healthy controls with a sensitivity and specificity of approximately 79%. Similarly, gas chromatography–ion mobility spectrometry (GC–IMS) and gas chromatography–time-of-flight mass spectrometry (GC–TOF-MS) have shown promising results in distinguishing pancreatic cancer patients from healthy individuals [29]. Collectively, these studies demonstrate that urine contains diverse classes of biomarkers reflecting proteolytic, proteomic, transcriptomic, and metabolic changes associated with pancreatic tumor development. A growing body of evidence supports the potential of urine as a valuable non-invasive source of biomarkers for the detection and monitoring of pancreatic cancer.

Taking the above into consideration, the URTESTE Research and Development Center (R&D Center URTESTE) Team initiated work on the development and analytical validation of a non-invasive, qualitative chromogenic urine test that detects proteolytic activity associated with pancreatic cancer. To capture proteolytic activity specifically associated with pancreatic cancer, three chromogenic peptide reagents are included in the device’s chemistry. These substrates are designed to undergo enzymatic cleavage by proteases associated with tumor-related proteolytic mechanisms, resulting in a measurable chromogenic signal. The assay therefore integrates biological relevance with a functional, activity-based measurement principle.

## 2. Materials and Methods

### 2.1. Chemistry

#### 2.1.1. Substrates and Solvents

All chemicals and solvents used for synthesis were purchased from commercial suppliers and applied directly in the experiments without further purification.

#### 2.1.2. Synthesis

**Scheme 1:**
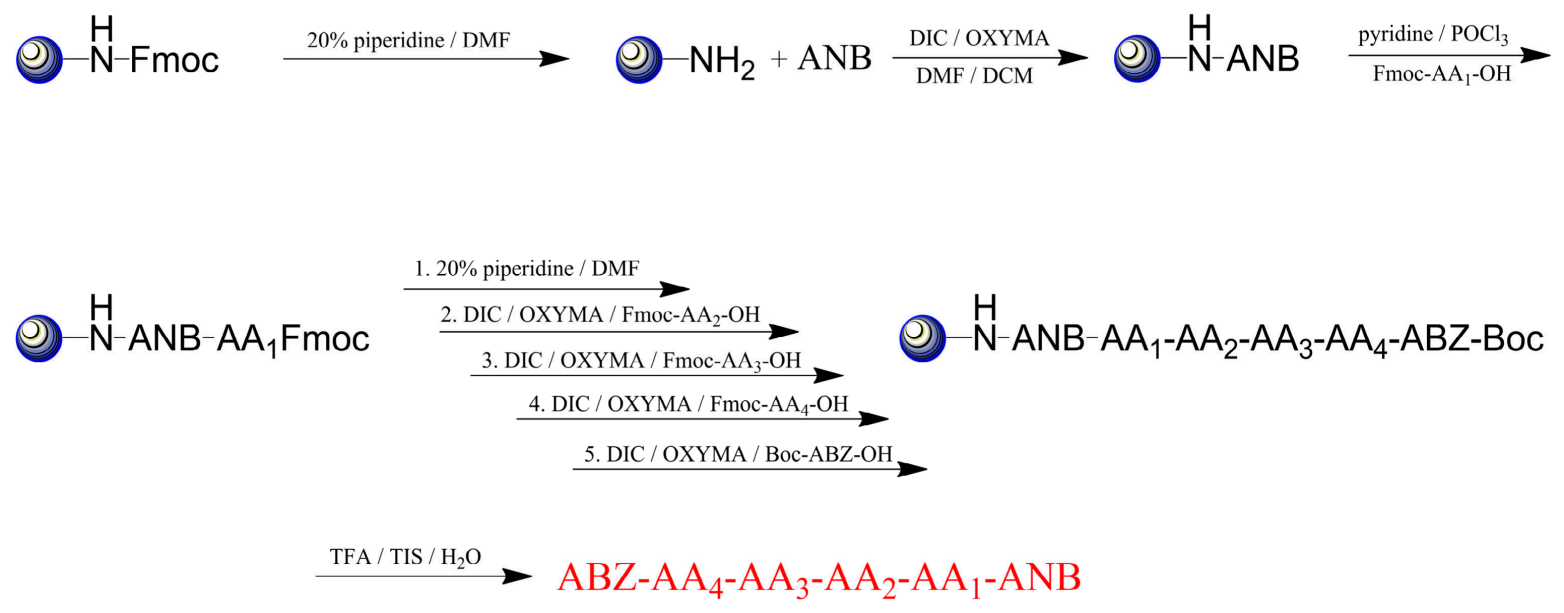
Synthetic route of reagents.

Three reagents were synthesized manually by the solid-phase Fmoc/tBu method, using Rink Amide resin (RAM resin; 0.63 mmol/g; Carbolution Chemicals GmbH, St. Ingbert, Germany) as a solid support. Deprotection of the Fmoc group was accomplished with 20% piperidine (Thermo Fisher Scientific, Waltham, MA, USA) in *N*,*N*′-dimethylformamide (DMF; Fisher Scientific, Waltham, MA, USA) for 20 min (Scheme 1).

The coupling of amino acids (Fmoc-AA-OH), 5-amino-2-nitrobenzoic acid (ANB; AmBeed, Buffalo Grove, IL, USA), and 2-Boc-aminobenzoic acid (Boc-ABZ-OH; ChemScene, Monmouth Junction, NJ, USA) was performed using a threefold excess of the reagents relative to the resin. The coupling mixture consisted of an equimolar ratio *N*,*N*′-diisopropylcarbodiimide (DIC; Iris Biotech, Marktredwitz, Germany), OxymaPure (Apollo Scientific, Manchester, UK), and the respective building block (Fmoc-AA-OH, ANB or Boc-ABZ-OH) dissolved in a DCM:DMF (4:1, *v*/*v*; Fisher Scientific, Waltham, MA, USA) solution, with agitation for 1.5 h. An exception was made for the coupling of the first amino acid to the ANB–RAM resin. For this purpose, the amino acid dissolved in pyridine (Fisher Scientific, Waltham, MA, USA) was added to the resin, the mixture was cooled to −20 °C, and POCl_3_ (Thermo Fisher Scientific, Waltham, MA, USA) was subsequently added. After completion of the reaction, the resin was washed with a sequence of solvents [DMF, DCM, methanol (VWR Chemicals, Radnor, PA, USA), and diethyl ether (Merck KGaA, Darmstadt, Germany)] and dried. The chloranil test (Merck KGaA, Darmstadt, Germany) was employed to monitor the progress of the coupling and deprotection steps. After the synthesis was completed, the reagents were cleaved from the resin using a mixture of trifluoroacetic acid (TFA; VWR Chemicals, Radnor, PA, USA), triisopropylsilane (TIS; Merck KGaA, Darmstadt, Germany), 1,3-dimethoxybenzene (Merck KGaA, Darmstadt, Germany), phenol (Merck KGaA, Darmstadt, Germany), and deionized water (90:2.5:2.5:2.5:2.5, *v*/*v*). The products were then precipitated with cooled diethyl ether and lyophilized.

Purification was performed using an preparative chromatograph (ECOM, Chrášťany, Czech Republic) equipped with an Uptisphere^®^ Strategy C18HQ HPLC column (250 × 21.2 mm, 100 Å, 10 µm; Advion Interchim Scientific, Montluçon, France). UV detection was conducted at 214 nm. The crude reagents were purified using a linear gradient of 5–40% acetonitrile (Th. Geyer GmbH & Co. KG, Renningen, Germany) over 30 min at a mobile phase flow rate of 30.0 mL/min. Both eluents, acetonitrile (ACN) and water, contained 0.1% (*v*/*v*) TFA. Pure fraction (>95% by HPLC) were collected and lyophilized. The purity and identity of the peptides were confirmed by LC–MS analysis using a Waters Alliance e 2695 system equipped with Waters 2998 PDA and Acquity QDA detectors (software: Empower 3 ver. FR5; Waters, Milford, MA, USA) and a HALO C18 column (4.6 × 100 mm, 2.7 µm particle size, 90 Å pore size; Advanced Materials Technology, Inc., Wilmington, DE, USA). Samples were analyzed using a 1–100% acetonitrile gradient in deionized water over 10 min at 25 ± 0.1 °C. The mobile phase flow rate was 0.5 mL/min, and both eluents contained 0.1% (*v*/*v*) formic acid (Merck KGaA, Darmstadt, Germany). Mass analysis and UV detection at 214 nm were utilized for characterization.

#### 2.1.3. Structure and Purity Determination

The purity and identity of the compounds were confirmed by LC–MS analysis using an RP-HPLC Waters Alliance e2695 system with Waters 2998 PDA and Acquity QDA detector (Empower 3 software ver. FR5; Waters Corporation, Milford, CT, USA). Elemental analysis (CHNS) was performed on a Flash 200 analyzer (Thermo Fisher Scientific, Waltham, MA, USA). ^1^H NMR spectra were recorded on a Varian Unity Inova 500 (500 Hz; Varian, Inc., Palo Alto, CA, USA).

### 2.2. Study Design

This study represents a single-center analytical validation of a prototype configuration of the Panuri qualitative in vitro diagnostic (IVD) assay, supplemented by a predefined three-site reproducibility assessment conducted to evaluate inter-laboratory variability. The analytical validation was performed in accordance with structured experimental designs aligned with CLSI EP05-A3 and EP07-A3 principles [30,31]. The validation program included cut-off determination, evaluation of diagnostic performance (sensitivity and specificity with corresponding 95% confidence intervals), precision assessment (within-run repeatability, within-laboratory precision, and inter-laboratory reproducibility), and interference testing using predefined microbiological and endogenous interferents. Reproducibility assessment was conducted across three independent laboratory sites using a standardized study protocol and a single reagent LOT to isolate site-related variance components. All analytical evaluations were performed using predefined interpretation criteria and a fixed three-reagent qualitative algorithm. Statistical analyses were descriptive in nature and were not intended for hypothesis testing or clinical outcome validation.

### 2.3. Study Population

The population was derived from a multicenter, non-interventional research study (PANURI_AV) conducted in Poland. Participants were prospectively enrolled across multiple clinical centers, including tertiary oncology and gastroenterology units.

The study included adult participants (≥18 years) assigned to one of four predefined cohorts based on clinical diagnosis: (1) patients with pancreatic ductal adenocarcinoma (PDAC), (2) patients with other malignancies, (3) patients with non-oncological diseases, and (4) healthy volunteers. Cohort allocation was performed by the investigator based on available diagnostic data and medical history, in accordance with the study protocol. Disease-specific inclusion and exclusion criteria were applied to ensure appropriate classification and to minimize confounding factors, including exclusion of patients with active urinary tract diseases and oncological comorbidities if applicable.

Urine samples were collected prospectively, according to a standardized protocol, across participating clinical sites and processed under predefined conditions prior to laboratory analysis. All samples were pseudonymized at the site level prior to transfer to the laboratory for analysis.

For preliminary cut-off determination and diagnostic performance evaluation, 142 urine samples were analyzed, including 60 samples from patients with PDAC (Cohort 1) and 82 samples from healthy volunteers (Cohort 4) (Table 1). In Cohort 1, 46 patients had their PDAC stage determined, including 7 with stage 1, 14 with stage 2, 9 with stage 3 and 16 with stage 4. Interference testing was performed using selected PDAC-positive and healthy samples. For each tested microorganism, six PDAC samples and six healthy samples were evaluated under baseline and interferent conditions.

The research study was conducted in accordance with the Declaration of Helsinki and Good Clinical Practice (GCP) guidelines. The research protocol (PANURI_AV) was reviewed and approved by the Bioethics Committee at the District Medical Chamber in Gdańsk, Poland (opinion No. 999/2023, issued on 24 October 2023). Any subsequent protocol amendments were submitted to and reviewed by the Bioethics Committee in accordance with applicable regulatory requirements. Written informed consent was obtained from all participants prior to inclusion in the study. The consent process covered participation in the research study, collection and analysis of biological material, and processing of personal data in accordance with applicable data protection regulations (GDPR).

### 2.4. Pre-Analytical Procedures

Urine samples were collected as first-morning midstream specimens according to predefined procedures aligned with the European Federation of Clinical Chemistry and Laboratory Medicine (EFLM) urinalysis guidelines. Samples were transported at 2–8 °C and processed within predefined time limits. Following initial verification, 99.99% glycerol was added to obtain a final concentration of approximately 10%, and samples were stored at −20 ± 3 °C for up to 28 days. Only samples subjected to a single freeze–thaw cycle were included. Samples were excluded if urinalysis revealed bilirubin (“moderate” or “large”), blood ≥ 10 cells/µL, protein ≥ 30 mg/dL, nitrite positive, leukocytes ≥ 15 cells/µL or Specific Gravity ≤ 1.005.

### 2.5. Panuri Assay Principle

The Panuri assay is based on enzymatic hydrolysis of synthetic chromogenic peptide substrates labeled with ABZ and ANB moieties. Cleavage of the substrate results in an increase in absorbance measured at 410 nm. Each urine sample was tested with three reagents (R1, R2, and R3) in triplicates. After blank subtraction, median absorbance values were calculated, and reaction kinetics were expressed as slope (linear regression) and OD/h values using Magellan 7.5 software. A sample was classified as positive if at least two of the three reagents produced OD/h values above their predefined cut-off; otherwise, the result was classified as negative. Measurements were performed at 37 °C for 2 h using a Tecan Infinite F Nano^+^ spectrophotometer (Tecan Group Ltd., Männedorf, Switzerland).

### 2.6. Cut-Off Determination

Preliminary cut-off values were established using urine samples from 60 PDAC patients and 82 healthy volunteers. Results were compiled in Microsoft Excel and evaluated across candidate threshold values. For each candidate cut-off, sensitivity and specificity were calculated based on qualitative classification. Final cut-offs for each reagent were selected to maximize sensitivity and specificity in the development dataset while enabling implementation of the result algorithm within the measurement software environment. Cut-off values (OD/h) applied in subsequent analyses were: R1 = 0.002206, R2 = 0.003343, and R3 = 0.000766. These values were applied for binary classification in subsequent analyses.

### 2.7. Diagnostic Performance

Diagnostic sensitivity (*Se*) and specificity (*Sp*) were calculated using the following formulas:

(1) $$Se=\frac{TP}{TP+FN}$$

(2) $$Sp=\frac{TN}{TN+FP}$$

*TP* = true positive; *TN* = true negative; *FN* = false negative; *FP* = false positive.

Sensitivity (*Se*) is defined as a percentage of samples correctly classified as having the condition. Specificity (*Sp*) is defined as a percentage of samples correctly classified as healthy.

Diagnostic accuracy (*DA*) was calculated using the following formula:

(3) $$DA=\frac{TP+TN}{TP+TN+FN+FP}$$

Diagnostic accuracy is defined as a percentage of correctly classified samples to an overall number of samples tested.

### 2.8. Precision (Repeatability and Reproducibility)

Precision of the Panuri assay was evaluated in accordance with CLSI EP05-A3 guidelines [22]. Precision studies were performed using the dedicated Panuri Control material, designed for internal quality control of the assay and representing predefined qualitative classification levels. The Panuri Control material was prepared, stored, and handled according to the manufacturer’s validation protocol to ensure stability and consistency throughout the precision evaluation.

#### 2.8.1. Repeatability (Single-Site Precision)

Repeatability and within-laboratory precision were assessed over 20 consecutive days, with two runs per day and two replicates per run across three reagent lots (20 × 2 × 2 × 3 design). The study was performed at a single site by one trained operator.

#### 2.8.2. Reproducibility (Multisite Precision)

Between-site reproducibility was evaluated across three independent laboratory sites over five days, with one run per day and five replicates per run (3 × 5 × 5 design). A single reagent lot was used at all sites. Variance components were estimated according to the EP05-A3 framework and expressed as coefficient of variation (CV%).

### 2.9. Interference Testing

Interference testing was conducted in accordance with CLSI EP07-A3 principles [31]. Both microbiological and non-microbiological interferents were evaluated. Microbiological interferents included Gram-positive and Gram-negative bacteria (*Escherichia coli* ATCC 8739, *Enterococcus faecalis* ATCC 29212, *Klebsiella pneumoniae* ATCC 10031, *Pseudomonas aeruginosa* ATCC 9027, and *Staphylococcus aureus* ATCC 6538) and a yeast (*Candida albicans* ATCC 10231). All microorganisms were tested at 10^7^ CFU/mL to represent worst-case contamination conditions. Non-microbiological interferents included albumin (60 g/L), bilirubin (0.12, 0.02, and 0.001 mg/mL), glucose (1000 mg/dL), and red blood cells (100,000 and 180,000/µL).

Further testing of exogenous substances will include antibiotics routinely applied for treatment of urinary tract infections, painkillers and substances commonly found in regular diet, such as glucose, caffeine and ascorbic acid.

For each interferent, paired analyses were performed using baseline (neat) urine samples and the same samples after addition of the interferent, as per the CLSI EP07 document [31]. Six PDAC-positive samples and six healthy control samples were included per microorganism. Results were classified as no change, false positive (FP), false negative (FN), or not evaluable (baseline misclassification). Interference was defined as a change in qualitative classification (positive ↔ negative). Only sample pairs with correct baseline classification were included in interference rate calculations.

### 2.10. Statistical Analysis

Diagnostic sensitivity and specificity were calculated for the selected cut-off values and reported with corresponding 95% confidence intervals. Analyses were descriptive in nature. No formal hypothesis testing was performed.

### 2.11. Determination of Panuri Assay Stability

Panuri stability was evaluated in accelerated conditions at 21 °C, 37 °C, in-use conditions (dissolved product stored at intended temperature), and at ambient temperature (dissolved product at room temperature 21 °C). All experiments were performed on 5 replicates of a selected representative enzyme as a reference material. All measurements of each replicate and each reagent were compared to first measurement as a reference point (T_0_). Median of OD/h was analyzed during these tests, along with an analysis of outliers. Acceptance criteria was ≤10% drift between reference point and the other points.

## 3. Results

### 3.1. Determination of Cut-Off Values and Diagnostic Performance

Preliminary cut-off values and diagnostic performance of the Panuri assay were established using urine samples from 60 patients with histologically confirmed PDAC (Cohort 1) and 82 healthy volunteers (Cohort 4). Two kinetic interpretation metrics were evaluated: total absorbance slope (ΔA/Δt) and optical density per hour (OD/h).

For each reagent (R1–R3), fixed reagent-specific cut-off values were determined during analytical performance evaluation and implemented in a predefined three-reagent binary algorithm. Positive (+) means readings above the cut-off and negative (−) means below the cut-off value for each reagent.

#### Comparison of Slope and OD/h Metrics

The diagnostic performance obtained using the slope and OD/h metrics is summarized in Table 2.

For the slope-based interpretation, sensitivity was approximately 88% (Table 3), specificity approximately 74%, and overall diagnostic accuracy approximately 80%.

For the OD/h-based interpretation, sensitivity was approximately 89%, specificity approximately 75%, and overall diagnostic accuracy approximately 80%.

The OD/h metric demonstrated slightly higher specificity while maintaining comparable sensitivity relative to the slope-based approach. Based on these results, OD/h was selected as the final interpretation parameter for subsequent analyses. The slope-based analysis is presented for comparative purposes only and was not used in subsequent validation analyses.

Across both interpretation approaches, the three-reagent algorithm provided balanced diagnostic performance, with sensitivity close to 90% and specificity in the range of 74–75%, supporting the suitability of the selected cut-off values for further validation studies.

### 3.2. Interference Testing and Analytical Robustness

Interference studies were conducted to assess the impact of microbial contamination and selected endogenous substances on Panuri test classification. Paired comparisons were performed between neat urine samples and urine samples spiked with predefined interferents. Only samples with correct baseline classification were included in interference rate calculations.

#### 3.2.1. Microbial Interference

Six microorganisms commonly associated with urinary tract infections were tested at a concentration of 10^7^ CFU/mL (worst-case scenario). For each microorganism, nine evaluable paired samples were analyzed after exclusion of baseline false-positive or false-negative cases. Interference, defined as a change in qualitative classification (positive ↔ negative), was observed in 4/9 to 5/9 evaluable pairs per microorganism, corresponding to interference rates of 44–56% (Table 4).

Interference predominantly manifested as false-positive shifts in samples from healthy volunteers (75–100% of evaluable negative samples), while false-negative shifts in PDAC-positive samples were less frequent (0–40%).

#### 3.2.2. Endogenous Interferents

The influence of albumin (60 g/L), bilirubin (0.12, 0.02, 0.001 mg/mL), glucose (1000 mg/dL), and red blood cells (100,000–180,000/µL) was evaluated. Misclassification events were observed sporadically across several interferents and concentrations (Table 5).

A substantial proportion of samples were classified as non-evaluable due to incorrect baseline results, reducing the number of valid paired comparisons. Within the available evaluable dataset, endogenous interferents demonstrated limited and concentration-dependent effects on Panuri classification.

### 3.3. Precision

Precision of the Panuri assay was determined following the CLSI EP05-A3 protocol [22]. Within-run repeatability and intermediate precision (within-laboratory) were evaluated using three independent reagent batches. Variance components attributable to between-run and between-day effects were calculated to characterize total analytical imprecision.

#### 3.3.1. Repeatability

Repeatability (within-run precision) ranged from 2.6% to 7.7% CV across all reagent batches.

#### 3.3.2. Within-Laboratory Precision

Within-laboratory precision (intermediate precision), including between-run and between-day variance components, is summarized in Table 6. The coefficients of variation (CV%) for the between-run and between-day components were generally low, indicating good stability of measurements across analytical runs and testing days. For Reagent R1, the between-day variability ranged from 0.0% to 2.5%, while the between-run variability ranged from 1.3% to 3.1% across the evaluated LOTs. The resulting intermediate precision (within-laboratory) remained consistent, with CV values between 4.1% and 4.2%. For Reagent R2, between-day variability ranged from 0.0% to 2.2%, and between-run variability from 0.0% to 2.6%. The corresponding within-laboratory precision ranged from 4.7% to 7.7% depending on the reagent LOT. For Reagent R3, slightly higher variability components were observed. The between-day CV ranged from 0.6% to 4.6%, while the between-run CV ranged from 3.3% to 7.1%. Consequently, within-laboratory precision values ranged from 6.7% to 9.7% across the tested LOTs.

#### 3.3.3. Reproducibility

Inter-laboratory reproducibility was assessed across three independent laboratories following the CLSI EP05-A3 [30] multi-site design and a single LOT of Panuri and Panuri Control chemistry. Reproducibility was measured daily for between-run/day, and subsequently, between-laboratory standard deviation (SD) and coefficient of variation (CV) were calculated. For Reagent 1, the between-site standard deviation (sss) was 0.0015 and a CV of 4.24%. For Reagent 2, the between-site SD was 0.0011 and a CV of 6.79%. For Reagent 3, the between-site SD was 0.0021 and a CV of 5.90%. Data are summarized in Table 7.

Grubb’s test was performed on datasets from all three sites to check for outliers, with g critical = 2.82 (α = 0.05). In the Reagent 1 dataset, the test returned a value of 2.8201 at Site 2, while in Reagent 2 Sites 1 and 2 datasets values of 2.8975 and 3.1880, respectively, were returned, indicating some isolated results in series.

### 3.4. Stability

Panuri showed accelerated stability for 400 days at 21 °C ± 3 °C (Table 8) and for 200 days at 37 °C ± 3 °C (Table 9). In the second case, it is assumed that high temperatures have a critical impact on the chemicals and confirmation of the stability must be set in real time conditions.

In-use stability (dissolved Panuri stored in 5° ± 3 °C) showed stability for 3 weeks (Table 10). There were more points tested, but Reagent 1 is less stable than others.

Ambient testing (dissolved product at room temperature 21 °C ± 3 °C) showed stability for 5 h (Table 11).

## 4. Discussion

This study presents the analytical validation of a prototype version of the Panuri qualitative urine assay developed for detection of PDAC-associated proteolytic activity. The validation was performed using structured experimental designs aligned with recognized CLSI performance evaluation frameworks (EP05-A3 and EP07-A3) [30,31], supporting methodological consistency with internationally accepted approaches to in vitro diagnostic (IVD) assay development.

### 4.1. Diagnostic Performance and Algorithm Design

Using established cut-off values and a three-reagent binary interpretation algorithm, the assay demonstrated 89% sensitivity and 75% specificity in the development cohort. The cut-off values were established within the development dataset and require independent validation in future studies. The OD/h metric provided slightly improved specificity compared to the slope-based interpretation while maintaining comparable sensitivity, and was therefore selected as the final interpretation parameter. The multi-reagent algorithm constitutes an important design element of the Panuri assay. By requiring concordant activation of at least two of three proteolytic substrates, the interpretation strategy reduces reliance on single-substrate variability and enhances classification robustness. This approach is particularly relevant for functional assays, where enzymatic activity may be influenced by biological heterogeneity. The achieved sensitivity supports the potential role of the assay as an adjunctive functional diagnostic tool in the evaluation of suspected PDAC. However, the moderate specificity observed in the development cohort indicates that results must be interpreted within clinical context. Given the functional, activity-based nature of the assay, the observed performance reflects detection of proteolytic processes rather than tumor-specific molecular signatures. The substrates were not designed to target individual PDAC-specific proteases, but rather to capture broader patterns of tumor-associated proteolytic activity that may occur in PDAC. This functional approach was selected to maximize detection of disease-associated proteolytic processes rather than individual molecular targets.

### 4.2. Interference and Pre-Analytical Boundary Conditions

Interference studies demonstrated that extreme microbial contamination (10^7^ CFU/mL) may induce classification shifts, predominantly false-positive results in initially negative samples. This concentration represents a worst-case contamination scenario and establishes defined pre-analytical boundary conditions for assay use. The findings emphasize the importance of standardized sample collection and exclusion of samples with significant urinary tract infection. The observed interference pattern suggests that active urinary tract infection or substantial microbial contamination may represent a potential confounding factor during result interpretation. As the interference was predominantly associated with false-positive classification shifts, results obtained from samples with suspected urinary tract infection should be interpreted with caution and in conjunction with other clinical findings. These observations support the implementation of appropriate pre-analytical controls and sample acceptance criteria when applying the assay in a clinical setting.

Endogenous interferents, including albumin, bilirubin, glucose, and red blood cells, demonstrated limited and concentration-dependent effects. Misclassification events were sporadic and occurred primarily at high or clinically extreme concentrations. Within the evaluable dataset, endogenous substances did not demonstrate systematic interference with assay classification. These results support acceptable analytical robustness under controlled pre-analytical conditions. Clear identification of interference boundaries is essential in early-stage assay development, as it informs risk management strategies and supports future conformity assessment under applicable regulatory frameworks.

### 4.3. Precision, Reproducibility, and Assay Transferability

Precision studies conducted in accordance with CLSI EP05-A3 [30] demonstrated repeatability ranging from 2.6% to 7.7% CV and within-laboratory precision ranging from 4.1% to 9.7% CV across three reagent batches. Inter-laboratory reproducibility evaluated across three independent sites showed between-site variability ranging from 4.24% to 6.79% CV. These values fall within acceptable ranges for enzymatic activity-based assays and indicate stable analytical performance at the prototype stage. Notably, between-site variability remained below 7% CV for all reagents, supporting assay transferability and operational consistency across laboratory environments. Given that the evaluated configuration represents a prototype device, the results provide preliminary evidence supporting lot-to-lot consistency under prototype production conditions. The use of a dedicated Panuri Control material further supports implementation of internal quality control procedures in subsequent development phases.

### 4.4. Diagnostic Use Boundaries (Rule-In/Rule-Out Context)

Considering the high sensitivity (89%) and moderate specificity (75%) demonstrated in the analytical validation, the Panuri assay may have potential as an adjunctive component of future diagnostic workflows. However, its clinical role cannot be established based solely on the present analytical validation and requires confirmation in independent prospective clinical performance studies. A positive result should be interpreted as an indicator of elevated proteolytic activity requiring further confirmatory evaluation, such as imaging or biopsy. Likewise, a negative result should not be considered sufficient to exclude PDAC in the absence of further clinical assessment. The assay is therefore not intended to be used as a standalone diagnostic test for PDAC, but rather as an adjunctive, functional assessment complementing existing clinical and imaging approaches.

### 4.5. Development Pathway and Regulatory Considerations

The present study represents analytical validation of a prototype configuration of the Panuri assay. While the results establish technical feasibility and defined analytical performance characteristics, additional development steps are required prior to regulatory conformity assessment. The next development phase will include production of three independent manufacturing batches under controlled production conditions, followed by comprehensive analytical performance verification and expanded clinical validation studies. These activities will be necessary to confirm batch-to-batch consistency, refine performance parameters where required, and fully support intended-use claims. The current findings therefore provide a structured analytical foundation for continued development of the Panuri assay as a qualitative IVD device designed to detect PDAC-associated proteolytic activity. Further evaluation of clinical performance is ongoing in a prospective clinical performance study compliant with ISO 20916 [32] and IVDR requirements.

## 5. Conclusions

The results demonstrate the technical feasibility of detecting PDAC-associated proteolytic activity in urine using a chromogenic substrate-based approach. However, several limitations should be considered when interpreting the findings. First, the assay remains a prototype under development, and the results presented here reflect development-phase investigations performed to support assay optimization and preliminary performance assessment. Consequently, these findings should not be considered equivalent to evidence generated through a formal clinical performance evaluation.

Second, the classification thresholds applied in this study were derived and evaluated using the same dataset, which may increase the risk of overfitting and lead to optimistic estimates of diagnostic performance. Therefore, the reported sensitivity and specificity should be regarded as preliminary.

Furthermore, the Panuri assay is not intended to function as a standalone diagnostic test but rather as an adjunctive tool supporting clinical decision-making in patients with suspected pancreatic cancer. Ongoing independent clinical validation studies are expected to provide a more comprehensive assessment of diagnostic accuracy, reproducibility, and clinical utility in representative patient populations. These studies will ultimately determine the extent to which the assay may contribute to future diagnostic pathways for pancreatic cancer.

## Figures and Tables

**Table 1:** Demographic characteristics of the study population.

Characteristic	PDAC (*n* = 60)	Healthy Controls (*n* = 82)
Age, years (median)	69	33
Female, *n* (%)	22 (36.7)	41 (50.0)
Male, *n* (%)	38 (63.3)	41 (50.0)

**Table 2:** Panuri device final result algorithm.

R1 Result	R2 Result	R3 Result	Final Call
+	+	+	positive
+	+	−	positive
−	+	+	positive
+	−	+	positive
−	−	+	negative
−	+	−	negative
+	−	−	negative
−	−	−	negative

**Table 3:** Diagnostic performance of the Panuri assay using slope and OD/h interpretation metrics.

Interpretation Metric	Sensitivity (%)	Specificity (%)	Overall Diagnostic Accuracy (%)
Slope	~88	~74	80%
OD/h	~89	~75	80%

**Table 4:** Microbial interference on Panuri test classification (10^7^ CFU/mL).

Microorganism	Cohort 1 (FN/*n*)	Cohort 4 (FP/*n*)	Total (FN + FP)/*n*
*Candida albicans*	2/5 (40%)	3/4 (75%)	5/9 (56%)
*Escherichia coli*	1/5 (20%)	4/4 (100%)	5/9 (56%)
*Enterococcus faecalis*	1/5 (20%)	4/4 (100%)	5/9 (56%)
*Klebsiella pneumoniae*	1/5 (20%)	4/4 (100%)	5/9 (56%)
*Pseudomonas aeruginosa*	0/5 (0%)	4/4 (100%)	4/9 (44%)
*Staphylococcus aureus*	1/5 (20%)	3/4 (75%)	4/9 (44%)

**Table 5:** Summary of endogenous interferent impact on Panuri classification.

Interferent	Cohort 1 (FN/*n*)	Cohort 4 (FP/*n*)	Total (FN + FP)/*n*
Albumin 60 g/L	0/1	1/1	1/2
Bilirubin 0.12 mg/mL	0/5	N/A	0/5
Bilirubin 0.02 mg/mL	N/A	2/2	2/2
Bilirubin 0.001 mg/mL	0/1	1/1	1/2
Glucose 1000 mg/dL	1/2	N/A	1/2
RBC 180,000/µL	1/2	0/1	1/3
RBC 100,000/µL	0/2	2/2	2/4

**Table 6:** Repeatability and within-laboratory precision results.

Reagent	LOT	sr (SD)	sr (CV%)	srr (SD)	srr (CV%)	sdd (SD)	sdd (CV%)	sRw (SD)	sRw (CV%)
Reagent 1	LOT 1	0.001191	3.7	0.000589	1.8	0.000000	0.0	0.001329	4.1
Reagent 2	LOT 1	0.001076	7.7	0.000000	0.0	0.000000	0.0	0.001076	7.7
Reagent 3	LOT 1	0.001871	5.8	0.001063	3.3	0.000188	0.6	0.002160	6.7
Reagent 1	LOT 2	0.000842	2.6	0.001024	3.1	0.000000	0.0	0.001326	4.1
Reagent 2	LOT 2	0.000502	4.0	0.000325	2.6	0.000232	1.8	0.000642	5.1
Reagent 3	LOT 2	0.001567	4.8	0.001623	5.0	0.000630	1.9	0.002342	7.2
Reagent 1	LOT 3	0.001094	3.1	0.000471	1.3	0.000868	2.5	0.001474	4.2
Reagent 2	LOT 3	0.000534	3.8	0.000215	1.5	0.000311	2.2	0.000655	4.7
Reagent 3	LOT 3	0.001572	4.8	0.002344	7.1	0.001525	4.6	0.003208	9.7

Abbreviation: sr—site repeatability; srr—site between-run repeatability; sdd—site between-day repeatability; sRw—within-laboratory precision.

**Table 7:** Inter-laboratory reproducibility (multisite study).

Reagent	sss (CV%)	sss (SD)
Reagent 1	4.24	0.0015
Reagent 2	6.79	0.0011
Reagent 3	5.90	0.0021

Abbreviations: sss—between-site reproducibility.

**Table 8:** Panuri accelerated stability at 21 °C testing results.

Timepoint 1–55 days		
Reagent	Calculated Drift	Result
R1	0.53%	PASS
R2 R3	−0.78% −5.21%	PASS PASS
**Timepoint 2–115 days**		
Reagent	Calculated Drift	Result
R1	−3.31%	PASS
R2 R3	−3.82% −7.99%	PASS PASS
**Timepoint 3–240 days**		
Reagent	Calculated Drift	Result
R1	−3.72%	PASS
R2 R3	−1.90% −1.85%	PASS PASS
**Timepoint 4–400 days**		
Reagent	Calculated Drift	Result
R1	−1.76%	PASS
R2 R3	−5.25% 4.50%	PASS PASS

**Table 9:** Panuri accelerated stability at 37 °C testing results.

Timepoint 1–60 days		
Reagent	Calculated Drift	Result
R1	−5.68%	PASS
R2 R3	3.76% −2.71%	PASS PASS
**Timepoint 2–200 days**		
Reagent	Calculated Drift	Result
R1	−2.27%	PASS
R2 R3	9.31% 1.35%	PASS PASS
**Timepoint 3–460 days**		
Reagent	Calculated Drift	Result
R1	−1.89%	PASS
R2 R3	−2.35% −12.22%	PASS FAIL

**Table 10:** Panuri in-use testing results.

Timepoint 1–1 week		
Reagent	Calculated Drift	Result
R1	1.03%	PASS
R2 R3	0.20% 4.16%	PASS PASS
**Timepoint 2–2 weeks**		
Reagent	Calculated Drift	Result
R1	−1.93%	PASS
R2 R3	−3.15% 1.93%	PASS PASS
**Timepoint 3–3 weeks**		
Reagent	Calculated Drift	Result
R1	−5.59%	PASS
R2 R3	−0.74% −0.31%	PASS PASS
**Timepoint 4–4 weeks**		
Reagent	Calculated Drift	Result
R1	−13.08%	FAIL
R2 R3	−2.15% −6.17%	PASS PASS

**Table 11:** Panuri ambient testing results.

Timepoint 1–1 h		
Reagent	Calculated Drift	Result
R1	−2.79%	PASS
R2 R3	−2.51% 0.28%	PASS PASS
**Timepoint 2–2 h**		
Reagent	Calculated Drift	Result
R1	−3.38%	PASS
R2 R3	−7.06% −0.81%	PASS PASS
**Timepoint 3–3 h**		
Reagent	Calculated Drift	Result
R1	−5.32%	PASS
R2 R3	−7.49% −2.93%	PASS PASS
**Timepoint 4–4 h**		
Reagent	Calculated Drift	Result
R1	1.08%	PASS
R2 R3	−4.02% 5.06%	PASS PASS
**Timepoint 5–5 h**		
Reagent	Calculated Drift	Result
R1	7.31%	PASS
R2 R3	−3.23% 9.31%	PASS PASS
**Timepoint 6–6 h**		
Reagent	Calculated Drift	Result
R1	−5.39%	PASS
R2 R3	−17.83% −2.17%	FAIL PASS

## Data Availability

The original contributions presented in this study are included in the article. Further inquiries can be directed to the corresponding author.

## Abbreviations

The following abbreviations are used in this manuscript:

ABZ	Aminobenzoic acid
ANB	5-amino-2-nitrobenzoic acid
ASCO	American Society of Clinical Oncology
ATCC	American Type Culture Collection
CA19-9	Carbohydrate Antigen 19-9
CEA	Carcinoembryonic Antigen
CFU	Colony Forming Unit
CHNS	Carbon, Hydrogen, Nitrogen, Sulfur
CI	Confidence Interval
CLSI	Clinical and Laboratory Standards Institute
CT	Computed Tomography
CV%	Coefficient of Variation
DA	Diagnostic Accuracy
DIC	*N*,*N*′-diisopropylcarbodiimide
DMF	*N*,*N*′-dimethylformamide
EFLM	European Federation of Clinical Chemistry and Laboratory Medicine
EGTM	European Group of Tumoral Markers
EUS	Endoscopic Ultrasound
EUS-FNA	Endoscopic Ultrasound Fine-Needle Aspiration
EUS-FNB	Endoscopic Ultrasound Fine-Needle Biopsy
FAIMS	Filed Asymmetric Ion Mobility Spectrometry
FDA	Food and Drug Administration
FN	False Negative
FP	False Positive
GC-IMS	Gas Chromatography-Ion Mobility Spectrometry
GC-TOF-MS	Gas Chromatography-Time-Of-Flight Mass Spectrometry
HPLC	High-Performance Liquid Chromatography
ICD	International Classification of Diseases
IVD	In Vitro Diagnostic
LC-MS	Liquid Chromatography-Mass Spectrometry
LYVE-1	Lymphatic Vessel Endothelial HA Receptor
miR	microRNA
MMPs	Matrix Metalloproteinases
MRI	Magnetic Resonance Imaging
NMR	Nuclear Magnetic Resonance
OD/h	Optical Density per hour
PDAC	Pancreatic Ductal Adenocarcinoma
QDA	Quadrupole Dalton
R	Reagent
RAM	Rink Amide Resin
REG1A	Regenerating Islet-Derived 1 Alpha
RP-HPLC	Reverse Phase High Performance Liquid Chromatography
SD	Standard Deviation
Se	Sensitivity
Sp	Specificity
TFA	Trifluoroacetic acid
TFF1	Trefoil Factor Family
TIS	Triisopropylsilane
TN	True Negative
TP	True Positive
UV	Ultraviolet
VOC	Volatile Organic Compound
